# New-Onset Collagenous Colitis in a Patient With Psoriatic Arthritis: Can It Be Secukinumab?

**DOI:** 10.7759/cureus.16147

**Published:** 2021-07-03

**Authors:** Adham E Obeidat, Traci T Murakami

**Affiliations:** 1 Internal Medicine, University of Hawaii, Honolulu, USA; 2 Gastroenterology and Hepatology, The Queen's Medical Center West Oahu, Ewa Beach, USA

**Keywords:** collagenous colitis, colitis, diarrhea, secukinumab, psoriatic arthiritis, arthritis

## Abstract

Collagenous colitis is a chronic inflammatory condition and one type of a bigger entity, microscopic colitis. Collagenous colitis is associated with autoimmune diseases, such as psoriatic arthritis, suggesting an immune mechanism involved in the pathogenesis of the disease. New onset and flares of inflammatory bowel disease have been associated with the use of secukinumab, but no cases of microscopic colitis have been reported yet. We present a case of a 41-year-old woman with psoriatic arthritis treated with secukinumab who developed chronic diarrhea and was found to have collagenous colitis.

## Introduction

Collagenous colitis is a chronic inflammatory condition affecting the colon and it is a distinct type of inflammatory bowel disease (IBD). It usually presents with chronic watery diarrhea, abdominal pain, and weight loss [1,2]. Collagenous colitis has been associated with the use of some medications, most commonly non-steroidal anti-inflammatory drugs (NSAIDs) [2,3]. Furthermore, many cases of collagenous colitis have been associated with autoimmune diseases including rheumatoid arthritis, systemic lupus erythematosus (SLE), celiac disease, and, less frequently, psoriatic arthritis [2,4,5]. Recently, development of new cases and flares of ulcerative colitis (UC) and Crohn’s disease (CD) have been associated with treatment with interleukin-17A (IL-17A) antagonist secukinumab, but to date, no cases of collagenous colitis have been reported [6-8]. Here, we report a case of a 41-year-old woman with psoriatic arthritis treated with secukinumab who was found to have collagenous colitis during an evaluation for chronic diarrhea.

## Case presentation

A 41-year-old woman with a past medical history of psoriatic arthritis, fibromyalgia, gastroesophageal reflux disease, and irritable bowel syndrome was referred to the gastrointestinal clinic for evaluation and management of chronic watery diarrhea. Three weeks before presentation, she had the onset of watery diarrhea, with no blood or mucus, which was recurring four times daily. Moreover, this was associated with waxing and waning abdominal pain, nausea, and unintentional weight loss of 3 kg in one week. The patient denied any vomiting, fever, chills, rigors, or urinary symptoms. A gluten-free and a BRAT (banana, rice, applesauce, and toast) diet did not help her symptoms. Patient’s physical examination was unremarkable including her abdominal exam.

She had an upper endoscopy done three years earlier for persistent epigastric pain. Histopathology showed mild chronic gastritis with a normal esophagus and duodenum. She reported significant improvement in her epigastric pain until she developed a persistent watery diarrhea. Patient started taking venlafaxine 15 years prior to onset of her diarrhea. She also took NSAIDs for two years and sulfasalazine intermittently. She started taking secukinumab nine months prior to the onset of her diarrhea.

**Figure 1:**
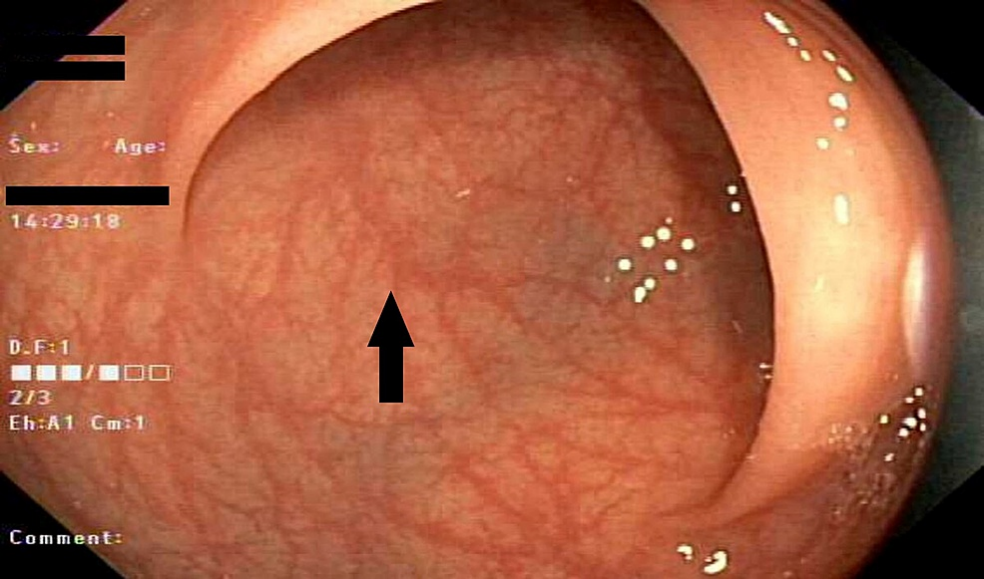
Colonoscopy image demonstrates moderate mucosal congestion. No polyps, nodules, or ulcerations.

**Figure 2:**
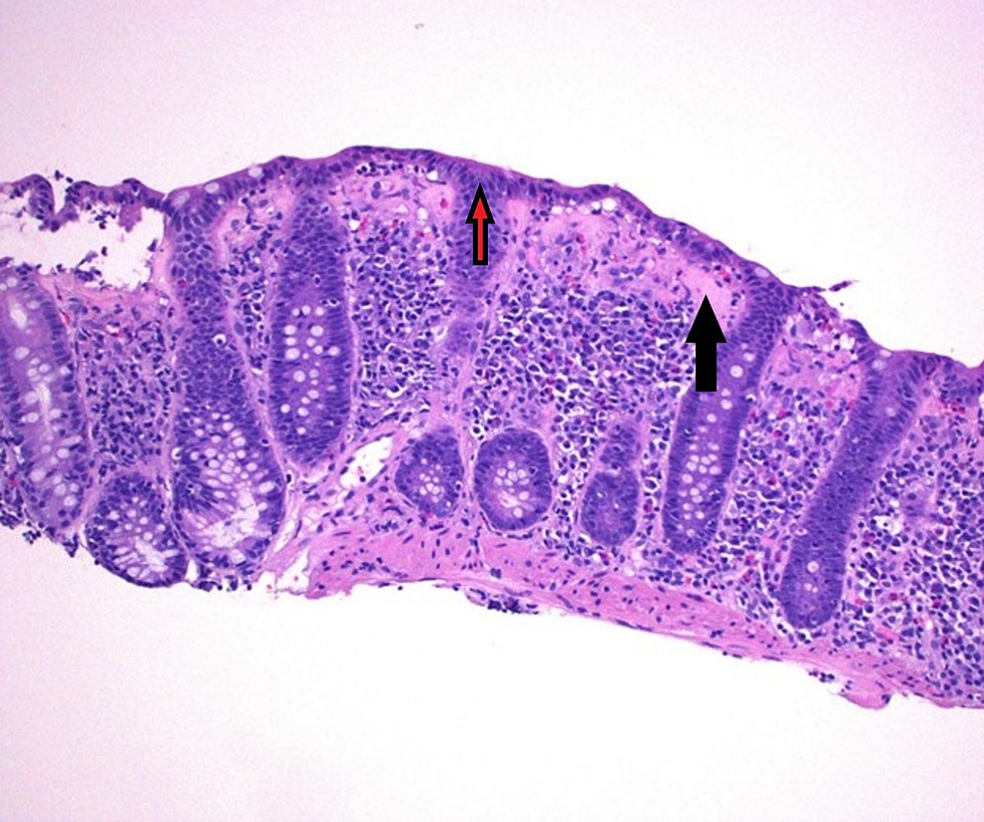
Microscopic image (H&E stain, 100x power and 200x zoom) showing thickening of sub-epithelial basement membrane (black arrow) along with increased intra epithelial lymphocytes (red arrow).

Blood and stool laboratory tests did not show any evidence of infection or malabsorption. Colonoscopy was done and it showed moderate mucosal congestion in transverse colon, ascending colon, and cecum, with non-bleeding internal hemorrhoids, but otherwise normal mucosa (Figure 1). Biopsies that were taken from multiple colonic sites and histopathological exam showed thickening of sub-epithelial basement membrane along with increased intraepithelial lymphocytes, which are consistent with the diagnosis of collagenous colitis (Figure 2). Immunological work-up including anti-nuclear antibodies, anti-centromere antibodies, anti-CCP antibodies, anti-snRNP antibodies, anti-Scl-70 antibodies, anti-SSA, and anti-SSB antibodies were all negative. The patient was started on budesonide with significant improvement in her symptoms. Budesonide was eventually tapered, and the patient was started on azathioprine as a steroid-sparing agent and immunomodulator to treat both collagenous colitis and psoriatic arthritis.

## Discussion

Microscopic colitis, which includes both collagenous colitis and lymphocytic colitis, is a chronic inflammatory condition of the colon, affecting females more than males and mostly people in their sixth or seventh decade of life [1,2,9]. The hallmark of collagenous colitis is chronic watery diarrhea. Collagenous colitis can present also with abdominal pain, weight loss, fecal incontinence, and bloody stool [2,9]. Laboratory and imaging studies are often normal, and the findings on colonoscopy are also normal. Diagnosis is made by performing biopsy and histopathological exam, which frequently shows diffuse thickening of the colonic sub-epithelial collagen layer, intraepithelial lymphocytosis, and infiltration of the lamina propria with inflammatory cells [1,2,5,10].

Collagenous colitis is associated with multiple autoimmune diseases including rheumatoid arthritis, Hashimoto’s thyroiditis, SLE, celiac disease, and spondyloarthropathies [2-4]. The first case of arthritis associated with collagenous colitis was described in 1983 by Erlendsson et al. [9]. Autoimmune disorders are associated with collagenous colitis in 20-60% of cases, and this association with autoimmune diseases raises the probability of an immune mechanism to be involved in the pathogenesis of the disease [2,4,5].

Collagenous colitis is associated with some medications as well, most frequently NSAIDs. The mechanism is unclear, but it is suggested that NSAIDs inhibit prostaglandin synthesis, which increases colonic mucosal permeability, and this will lead to more intraluminal fluid, which in turn leads to increased risk of infection and inflammation. The length of NSAIDs use before the onset of symptoms of microscopic colitis can vary between few weeks to 15 years, and most people resolve directly after stopping the medication although some may need few months [3]. Our patient was taking NSAIDs intermittently for two years prior to onset of her diarrhea, but given the lack of improvement although she stopped using this medication, it is unlikely that it contributed to her symptoms. Other medications associated with the development of collagenous colitis include selective serotonin reuptake inhibitors, statins, and proton pump inhibitors, but the mechanism is unclear and most of the data came from descriptive and uncontrolled studies [3,11,12]. 

The relationship between the use of secukinumab and the development of IBD is controversial. Some cases of UC and CD were reported in patients treated with secukinumab for various rheumatological diagnoses including ankylosing spondylitis, psoriatic arthritis, and plaque psoriasis. Duration of treatment varied between two and eight weeks prior to onset of symptoms [6,8]. On the other hand, two large studies were done to assess the efficacy of IL-17A antagonist in treating spondyloarthropathies, but they found no difference in the incidence of developing a new diagnosis or flares of IBD compared to placebo [13,14]. Moreover, two meta-analyses were conducted and they showed no increased risk of IBD with secukinumab in patients treated for ankylosing spondylitis, psoriasis, or psoriatic arthritis [15,16].

The consideration of secukinumab as a potential trigger of IBD started in 2012 when Hueber et al. reported that secukinumab has not proven to be effective in treating active CD cases, and unexpectedly, there was an increased incidence of adverse events compared to placebo, which suggested a possible protective function of IL-17A in patients with CD [7]. Although causality has not been established yet, clinicians should be aware of the potential side effects of secukinumab. The mechanism of how secukinumab can lead to the development of IBD is unclear, but a protective function of IL-17A in maintaining intestinal barrier integrity has been described [17,18]. Furthermore, one study showed that neutralizing IL-17 enhanced the development of dextran sulfate sodium-induced colitis in mice by increasing tumor necrosis factor-alpha, interferon-gamma, and IL-6, which can promote the inflammatory response [19].

## Conclusions

Our patient's symptoms started few months after starting secukinumab for the treatment of her psoriatic arthritis. Although a few cases of CD and UC associated with secukinumab use have been reported in the literature, this is the first case of collagenous colitis to be reported. Another possibility is that the patient had an undiagnosed collagenous colitis, and an acute flare has been triggered by the use of secukinumab. Although secukinumab is a safe and effective drug in treating psoriasis, psoriatic arthritis, and ankylosing spondylitis, it can be associated with rare but significant adverse events, which may lead to the development and trigger the flare of any of the spectrum of IBD from microscopic colitis to CD or UC, so physicians should be aware of these potential adverse events before starting the treatment.
